# Transcranial 1064-nm laser photobiomodulation modulates frequency-specific cortical source dynamics and functional connectivity in healthy adults

**DOI:** 10.3389/fnhum.2025.1704482

**Published:** 2026-01-08

**Authors:** Subrat Bastola, Tyrell Pruitt, Elizabeth M. Davenport, Joseph A. Maldjian, Hanli Liu, George Alexandrakis

**Affiliations:** 1 Department of Bioengineering, University of Texas at Arlington, Arlington, TX, United States; 2 Department of Radiology, UT Southwestern Medical Center, Dallas, TX, United States

**Keywords:** cortical network dynamics, directional connectivity, EEG-MEG, neuromodulation, phase-amplitude coupling, source localization, transcranial photobiomodulation

## Abstract

**Introduction:**

Transcranial photobiomodulation (tPBM) with near-infrared light is a promising non-invasive method to enhance cognition and support brain health. However, its mechanistic effects on large-scale cortical dynamics remain poorly understood. Establishing how tPBM reorganizes oscillatory hierarchies is critical for advancing both neuroscience and clinical translation.

**Methods:**

We examined whether acute 1,064-nm tPBM modulates oscillatory power, dipole source trajectories, and functional connectivity in the human brain. Simultaneous magnetoencephalography (MEG) and electroencephalography (EEG) were recorded in 25 healthy adults before and after prefrontal tPBM. Distributed source imaging (sLORETA) and global optimization dipole modeling characterized spatiotemporal alpha and beta activity. Connectivity was assessed with phase transfer entropy, and infra-slow phase–amplitude coupling analyses assessed hierarchical modulation.

**Results:**

Transcranial photobiomodulation induced frequency-specific reorganization of cortical networks. Alpha oscillations engaged coordinated fronto–visual circuits, whereas beta activity preferentially recruited higher-order executive regions. Source imaging revealed a post-stimulation shift from default mode toward central executive network dominance with stronger directed interactions. Infra-slow rhythms (<0.1 Hz), encompassing both very-low-frequency (0.01–0.1 Hz) and ultra-slow (<0.01 Hz) activity, significantly modulated alpha- and beta-band amplitudes, embedding faster oscillations within slower temporal patterns.

**Discussion:**

The findings of his work indicate that tPBM influences intrinsic brain activity by reorganizing oscillatory patterns and shifting network engagement. The redistribution from default mode toward executive systems, along with the nesting of faster rhythms within slower temporal structures, reflects a capacity for large-scale functional rebalancing. The results highlight tPBM’s potential as a precision neuromodulation tool for modulating executive and cognitive control systems.

## 1 Introduction

Transcranial photo-biomodulation (tPBM) is a non-invasive neuromodulation technique that modulates neural function through bioenergetic rather than direct electrophysiological mechanisms. Unlike electrical (transcranial direct current stimulation) (Galli et al., 2019; Mancuso et al., 2016; Brasil-Neto, 2012) or magnetic (transcranial magnetic stimulation) (Coffman et al., 2014; Grafman and Wassermann, 1998) approaches, which directly perturb membrane potential, tPBM enhances mitochondrial function via near-infrared (NIR) light absorption by cytochrome c oxidase (CCO) (Wang et al., 2019; Pruitt et al., 2020). This primary interaction triggers nitric oxide release, vasodilation, and increased cerebral blood flow (Ren et al., 2022; Toda et al., 2009) thereby boosting cellular energetics, reducing oxidative stress, and supporting neuroplasticity basis for its reported cognitive and therapeutic benefits (Saucedo et al., 2021; Gonzalez-Lima, 2021).

The efficacy of tPBM critically depends on wavelength selection. While CCO exhibits absorption peaks in the red to shorter NIR spectrum (660–810 nm), longer wavelengths around 1,064 nm offer superior penetration depth, up to 3–4 cm into brain tissue-due to reduced scattering and lower absorption by hemoglobin and water (Henderson, 2024; Hamblin, 2024). This has established 1,064 nm as the preferred wavelength for targeting prefrontal regions linked to executive control and attention (Vieira et al., 2023). Human studies confirm increased oxidized CCO and cerebral oxygenation, and randomized controlled trials demonstrate improvements in working memory, reaction time, and executive function following 1,064 nm tPBM (Hamblin, 2024; Zhang et al., 2025). Prior work from our co-author’s lab has examined the metabolic and hemodynamic consequences of tPBM using broadband and functional near-infrared spectroscopy at 800, 850, and 1,064 nm, demonstrating wavelength-dependent increases in cortical oxygenation and oxidized CCO levels (Tian et al., 2016; Pruitt et al., 2020; Shahdadian et al., 2023).

Likewise, EEG investigations show frequency-specific effects, with tPBM enhancing alpha (8–12 Hz) and beta (12–30 Hz) power while reducing slower rhythms (Wang et al., 2019, 2021). These findings suggest selective modulation of cortical excitability, yet conventional EEG analyses averaging pre/post-power fail to capture dynamic spatiotemporal reorganization (Kaur et al., 2022). Moreover, EEG’s limited spatial resolution and volume conduction confound localization of neural generators (Eom, 2022; Nielsen et al., 2023), leaving uncertainty about whether tPBM primarily influences Brodmann areas (BAs) or functional networks such as the default mode network (DMN; BAs 10, 23, 24, 31, 39) and the central executive network (CEN; BAs 9, 46, 40) (Daigle et al., 2022; Menon, 2023).

Magnetoencephalography (MEG) overcomes these limitations with superior spatial resolution, millisecond temporal precision, and reduced volume conduction (Hari and Puce, 2023). Despite this, only two studies have applied MEG to tPBM to date (Pruitt et al., 2024; Pruitt et al., n.d.). MEG combined with structural MRI enables precise mapping of oscillatory changes to cortical regions and BAs (Noguchi-Shinohara et al., 2021), and equivalent current dipole (ECD) modeling can track neural activity trajectories with high temporal resolution (Eom, 2022; Michel and He, 2019). Unlike distributed source methods that blur activations (Thio et al., 2023), dipole models resolve discrete generators over time, making them well-suited for studying propagation patterns following stimulation (Orsher et al., 2023). Global optimization algorithms further improve source localization, outperforming conventional least-squares methods prone to converging to local minima (Opoku et al., 2021; Ocak, 2008; Damayanti and Pratiwi, 2016) with demonstrated accuracy in both simulated and empirical MEG/EEG data (Bastola et al., 2024; Khosla et al., 1997).

Both EEG and MEG have been used to map neural oscillations, which provide the temporal structure that underlies perception, attention, and higher-order cognition by rhythmically organizing neuronal excitability and coordinating communication across distributed brain networks (Başar et al., 2001; Jensen and Mazaheri, 2010). These rhythms support behavior through mechanisms such as phase-dependent gating, synchronization, and large-scale integration, and their functional relevance is reflected in frequency-specific associations with sensory, motor, and cognitive operations (Siebenhühner et al., 2016). Prior studies have shown alpha and theta activity governs attentional sampling and working-memory maintenance (Khader et al., 2010; Klimesch, 2012), while beta oscillations regulate motor planning and sensorimotor integration (Brovelli et al., 2004). Accordingly, fluctuations in these rhythms are strongly associated with variations in perceptual performance, cognitive effort, and motor behavior (Jensen and Mazaheri, 2010; Schmidt et al., 2019). Because oscillatory dynamics arise from metabolically dependent membrane and synaptic processes, they can be externally modulated by noninvasive stimulation-including tPBM, which alters neuronal energetics, nitric oxide signaling, and vascular tone (Kashiwagi et al., 2023; Yan et al., 2025). Recent studies report frequency-specific modulation in task-relevant bands, suggesting that tPBM-induced metabolic shifts can translate into measurable changes in both neuronal activity and associated behavioral performance (Pruitt et al., 2020; Wang et al., 2021).

Oscillations themselves follow hierarchical roles: alpha supports top-down inhibition and internal attention, while beta stabilizes sensorimotor and cognitive states (Schmidt et al., 2019; Khanna and Carmena, 2015). These rhythms align with functional networks, with alpha linked to DMN activity at rest and beta supporting CEN control processes (Li et al., 2017; Bowman et al., 2017). Connectivity analyses are essential for understanding how tPBM reorganizes such networks. While coherence measures indicate synchronization, they cannot capture directionality; phase transfer entropy (PTE) offers a model-free solution for quantifying causal influences (Vicente et al., 2011; Thatcher, 2012; Ursino et al., 2020). Similarly, phase-amplitude coupling (PAC) reveals cross-frequency interactions where slower rhythms modulate faster oscillations (Molaee-Ardekani et al., 2010; Richter et al., 2017; Väyrynen et al., 2023; Mäki-Marttunen et al., 2025) providing a temporal scaffold for large-scale coordination. Whether tPBM influences PAC remains unexplored (Truong et al., 2022). Resting-state paradigms are particularly sensitive for detecting tPBM-induced reorganization, as they capture intrinsic network activity without task constraints (Väyrynen et al., 2023; Fox and Raichle, 2007). Although analysis is challenged by non-stationarity and the absence of event-locked averaging (Kim et al., 2023), advanced time-frequency and source modeling approaches can reveal spatiotemporal patterns otherwise hidden in spontaneous activity. Yet, such advanced multimodal, source-resolved analyses have not been systematically applied to tPBM, leaving a critical gap in our ability to characterize how the intervention reorganizes large-scale cortical networks and to leverage these dynamics for optimizing stimulation targets and dosing.

Here, we employ concurrent MEG-EEG with advanced electromagnetic source imaging to characterize 1064 nm tPBM effects on resting-state neural dynamics. By combining distributed source imaging to map cortical activations with global-optimization ECD modeling to trace dipole trajectories, we delineate how oscillatory activity propagates across Brodmann areas and functional networks. These findings help establish mechanistic foundations for optimizing tPBM interventions in cognitive enhancement and neurotherapeutics for disorders linked to dysregulated neural oscillations.

## 2 Materials and methods

### 2.1 Participants

Twenty-five healthy adult volunteers (mean age = 25.6 ± 4.8 years; 13 females, 12 males) participated in this study. All participants had normal or corrected-to-normal vision, and no self-reported history of neurological or psychiatric disorders. Written informed consent was obtained from each individual in accordance with a study protocol approved by the Institutional Review Board (IRB) of the University of Texas Southwestern Medical Center (UTSW). All recordings were conducted at the UTSW Magnetoencephalography (MEG) Center.

### 2.2 Experimental design

**FIGURE 1:**
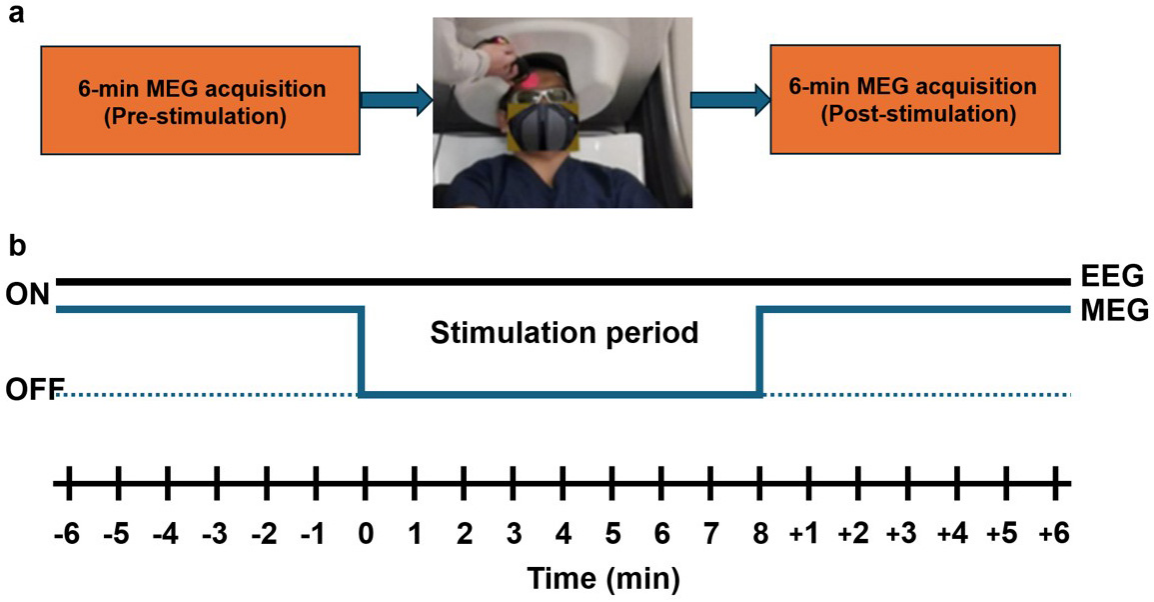
**(a)** Experimental protocol for magnetoencephalography (MEG) data acquisition consisting of a 6-min pre-transcranial photobiomodulation (tPBM) MEG recording, 8-min tPBM stimulation applied to the right forehead outside the MEG helmet, and a subsequent 6-min post-tPBM MEG recording. **(b)** Timeline of electroencephalography (EEG) and MEG acquisition sequences across the 20-min session. EEG was recorded continuously throughout the pre-, during-, and post-stimulation periods, whereas MEG was acquired only during the 6-min pre- and 6-min post-stimulation intervals.

Each subject underwent two experimental sessions (active tPBM and sham) conducted on separate days. Each session included three distinct recording phases. The first phase consisted of a 6-min pre-stimulation baseline with resting-state eyes-open recording using simultaneous MEG and EEG acquisition (Figure 1). Participants were instructed to fixate on a visual cross without engaging in any specific task. This was followed by an 8-min stimulation phase during which either active tPBM or sham stimulation was administered. During this stimulation period, only EEG data were recorded due to electrical interference from laser operation. The final phase involved a second 6-min post-stimulation resting-state eyes-open recording that was identical to the pre-stimulation phase, again with simultaneous MEG and EEG acquisition.

All sessions were conducted in a magnetically shielded room, and participants were monitored continuously for motion and discomfort. Head position was realigned after the stimulation phase to match the initial pre-stimulation alignment using real-time head localization.

#### 2.2.1 tPBM and sham stimulation protocol

Transcranial photobiomodulation (tPBM) was delivered using a continuous-wave 1,064-nm Class IV laser system (Model CG-5000, Cell Gen Therapeutics, Dallas, TX), a device that has received FDA clearance for clinical applications including pain reduction and inflammation management (Wang et al., 2019). While the FDA clearance pertains to somatic conditions, the wavelength and fluence parameters of this device have also been adapted in research targeting neuromodulatory effects on cortical function (Pruitt et al., 2020). The device featured a circular aperture with a diameter of 4.2 cm, delivering a uniform power density of 250 mW/cm^2^. This resulted in a total delivered fluence of approximately 107 J/cm^2^ over the 8-min stimulation duration, reflecting the measured effective irradiance (∼223 mW/cm^2^) after optical and diffuser losses.

The laser beam was directed at the right prefrontal cortex, approximately 1 cm below the Fp2 electrode position (International 10–10 system), a region implicated in executive function, attentional control, and known to exhibit tPBM-induced changes in prior studies (Barrett and Gonzalez-Lima, 2013; Wang X. et al., 2016). To accommodate the physical constraints of the MEG system, participants were seated in a reclined position such that their forehead extended below the MEG helmet during stimulation. Both participants and study personnel wore protective goggles with optical density (OD) ratings of 5+ at 900–1,000 nm, 7+ at 1,000–2,400 nm, and 7+ at 2,900–10,600 nm.

For the sham condition, the same device and setup were used, but the aperture was occluded with a solid 5-mm-thick opaque 3D-printed black PLA cap to block transmission of the 1,064-nm beam fully. The device remained powered on at its lowest setting (0.1 W) to match the characteristic hum of the device when powered on, preserving single-blind masking. Participants wore protective laser goggles, and the device was positioned out of view, preventing visual cues. Additionally, the snug fit of the EEG cap distributes uniform pressure across the forehead, reducing focal tactile contrast and making subtle warmth more difficult to perceive. Prior studies using similar irradiance levels show that acute irradiance less than 750 mW/cm^2^ for under 10 min tPBM produces a < 1 °C scalp temperature increase (Zein et al., 2018), which is minimal as a thermal perturbation and generally below the perceptual threshold of heat (Wang et al., 2021; Nairuz et al., 2024). Both tPBM and sham sessions followed identical experimental timelines and were counterbalanced across participants to mitigate order effects. *Post hoc* power-meter measurements confirmed no detectable transmitted irradiance beneath the 5-mm PLA cap (∼3 μW/cm^2^ noise floor), indicating the sham condition received effectively zero light. Finally, after both sessions, the participants were led to an MRI scanner for T1-weighted MRI scans, which were utilized to co-register with the anatomical fiducials marked for MEG sensors.

#### 2.2.2 MEG and EEG data acquisition and analysis

Simultaneous MEG-EEG recordings were acquired using a whole-head MEGIN Neuromag TRIUX Neo system (Helsinki, Finland) comprising 102 magnetometers and 204 planar gradiometers, and a 64-channel Easycap (Easycap GmbH, Germany) with electrodes positioned according to the international 10–10 configuration (Pruitt et al., 2024). All data were sampled at 1,000 Hz with online anti-aliasing filters, and EEG electrode impedances were maintained below 5 kΩ throughout recording. Precise spatial co-registration was achieved through five head position indicator (HPI) coils integrated into the EEG cap, enabling continuous head position tracking relative to MEG sensors (Medvedovsky et al., 2007). Anatomical fiducials (nasion and bilateral preauricular points), EEG electrode positions, HPI coil locations, and 300 additional scalp points were digitized using a Polhemus FASTRAK 3D digitizer to facilitate accurate co-registration with the MNI152 MRI template.

Electroencephalography and MEG data were first imported into Brainstorm (Tadel et al., 2011) for further analysis. A constant DC offset was removed, and notch filters were applied at 60 Hz and 120 Hz to eliminate line noise. Data were then band-pass filtered between 0.5 and 150 Hz using a zero-phase Butterworth filter. Physiological artifacts were attenuated using a two-stage approach. Signal-space projection (SSP) was used to identify and remove cardiac and ocular artifacts (Ille et al., 2002; Langlois et al., 2010). The preprocessed data were downsampled to 500 Hz to reduce computational demands while preserving sufficient temporal resolution for analysis of alpha, beta, and infra-slow oscillatory dynamics.

For source modeling, each subject’s MEG–EEG data were co-registered to their individual T1-weighted MRI, and a three-layer boundary element model (BEM) was computed in Brainstorm implementation to serve as the forward model for source reconstruction (Medani et al., 2023). Distributed source imaging was performed using standardized low-resolution electromagnetic tomography (sLORETA) on the subject-specific cortical surface (5-mm resolution) (Evans et al., 2012). The forward solution was computed based on the BEM model, and the inverse solution used an identity matrix as the noise covariance matrix, consistent with sLORETA’s assumption-free framework (Pascual-Marqui, 2002). To characterize large scale network change pre- and post tpbm, an additional gSVD-ICA neural source isolation was applied at the subject level, and the top five components accounting for more than 75% of the total variance and exhibiting consistent spatial patterns across subjects were retained. These components were transformed into MNI space and averaged separately for the pre-tPBM and post-tPBM conditions. sLORETA was then applied to estimate the corresponding cortical generators. Statistical significance was assessed using non-parametric permutation testing (Unseld et al., 2025) with false-discovery-rate correction, and only regions with corrected *p* < 0.05 were displayed. Dipole fitting was likewise performed on each subject’s individual MRI using the same forward model. For group analysis, both the subject-level sLORETA solutions and dipole coordinates were non-linearly normalized and projected onto the MNI152 template, allowing all participants’ data to be represented within a common anatomical space. sLORETA was applied separately to pre- and post-stimulation epochs for each subject to estimate cortical current density before transformation to MNI space.

To resolve focal source dynamics, equivalent current dipole (ECD) modeling was performed using a simulated annealing based global optimization algorithm, followed by a local parameter search (Bastola et al., 2024). The continuous time series data were segmented into 500 ms windows, with the algorithm searching for up to two dipoles (one per hemisphere) per window that minimized the difference between measured and modeled sensor fields for different frequency bands alpha (8–12 Hz), beta (12–30 Hz). The optimization algorithm consisted of a coarse simulated annealing-based global search followed by local gradient descent. Dipoles with goodness-of-fit (GOF) greater than 60% were retained (Mai and Majtanik, 2017), then projected onto the cortical surface for spatiotemporal mapping. For group-level analyses, subject-level dipoles corresponding to the same time windows were pooled, and only those falling within <10 mm of the dipole centroid (Almubarak et al., 2014) were retained and visualized on the MNI template. Validation of dipole solutions was performed by assessing anatomical plausibility, consistency with known functional regions, and recurrence across time windows. All subsequent statistical and visualization analyses were conducted using MATLAB (R2024a), Brainstorm (updated Nov 2024), and Fieldtrip (updated Nov 2024) (Oostenveld et al., 2011).

### 2.3 Statistical analysis

Group-level differences in Phase Transfer Entropy (PTE) connectivity between tPBM and sham were assessed using paired-samples *t*-tests as the primary test statistic. Statistical significance was established using a 5,000-iteration permutation procedure, in which condition labels were randomly sign-flipped within subjects to generate an empirical null distribution of mean PTE differences. The resulting *p*-values were corrected for multiple comparisons using Benjamini–Hochberg FDR (*q* < 0.05). For all significant connections, effect sizes (Cohen’s d) (Van Schependom et al., 2014; Keyvanfard et al., 2023) were computed, with particular attention to physiologically meaningful pathways in the alpha and beta bands.

Phase–amplitude coupling (PAC) between infra-slow (<0.1 Hz) phases and alpha/beta amplitudes was statistically assessed using a surrogate-shuffle method. Amplitude envelopes were circularly shifted to create a null distribution of modulation indices, from which z-scored MI values were derived. Only bins exceeding (*p* < 0.05) were retained, providing statistical confirmation of the observed infra-slow modulation cycles (∼0.03–0.05 Hz).

Dipole solutions were retained when the goodness-of-fit (GOF) exceeded 60% (Tamilia et al., 2019; Alhilani et al., 2020; Ntolkeras et al., 2022); however, additional validation was performed to address potential variability. To assess correspondence with sLORETA sources, a fraction-overlap metric was calculated by comparing dipole cluster locations with statistically significant sLORETA grids (Schwarzkopf et al., 2012) (obtained using non-parametric permutation testing with whole-brain FDR control). The observed overlap was evaluated against a null distribution generated by random spatial rotations within the cortical mask.

All sLORETA maps were statistically evaluated using the statistical non-parametric mapping (SnPM) framework (5,000 permutations, FDR-corrected *q* < 0.05). Furthermore, to assess large-scale network organization before and after tPBM, ICA was applied to retain meaningful neural components prior to source localization. Group level Brodmann areas showing FDR-significant sLORETA activations pre- and post-tPBM were extracted as virtual electrodes, and amplitude-envelope correlation (AEC) was computed among all ROI pairs. Louvain modularity was then used to derive community structure (Glomb et al., 2020; Sporns et al., 2021; Singlan et al., 2025), and the procedure was repeated 1,000 times to generate co-assignment indices quantifying the stability of ROI community membership across runs. Analyses were performed in MATLAB R2024a, Fieldtrip, and Brainstorm.

## 3 Results

**FIGURE 2:**
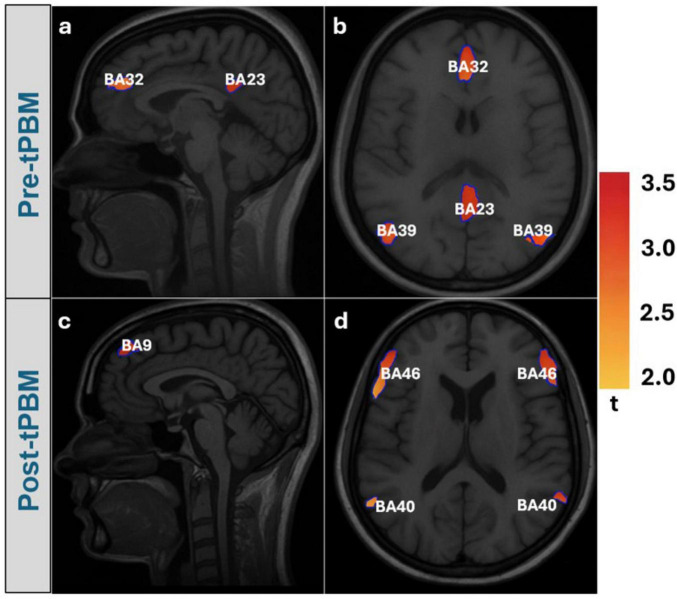
Standardized low-resolution electromagnetic tomography (sLORETA) source localization reveals network reorganization following transcranial photobiomodulation (tBPM): Sagittal and axial views of cortical activation from the top five independent components overlaid on the MNI152 template with Brodmann areas. **(a,b)** pre-tPBM shows Default Mode Network (DMN) activation (BA9, 23, 31, 32). **(c,d)** post-tPBM demonstrates a suggestive shift toward Central Executive Network (CEN) regions (BA10, 32, 39, 31, 23).

Figure 2 presents sagittal and axial views of the group-level cortical activations derived from Group Singular value Decomposition (gSVD) based Independent Component Analysis (ICA) (Anderson et al., 2006) and sLORETA analysis. In the pre-tPBM condition (Figures 2a,b), significant activation was observed in BA32 (anterior cingulate cortex), BA23 (posterior cingulate cortex), and bilateral BA39 (angular gyrus) areas commonly associated with DMN activity (Raichle, 2015). In the post-tPBM condition (Figures 2c,d), significant clusters appeared in BA9 (dorsolateral prefrontal cortex), bilateral BA46 (middle frontal gyrus), and BA40 (supramarginal gyrus) areas commonly associated with CEN activity (Bernal et al., 2016; Daigle et al., 2022).

**FIGURE 3:**
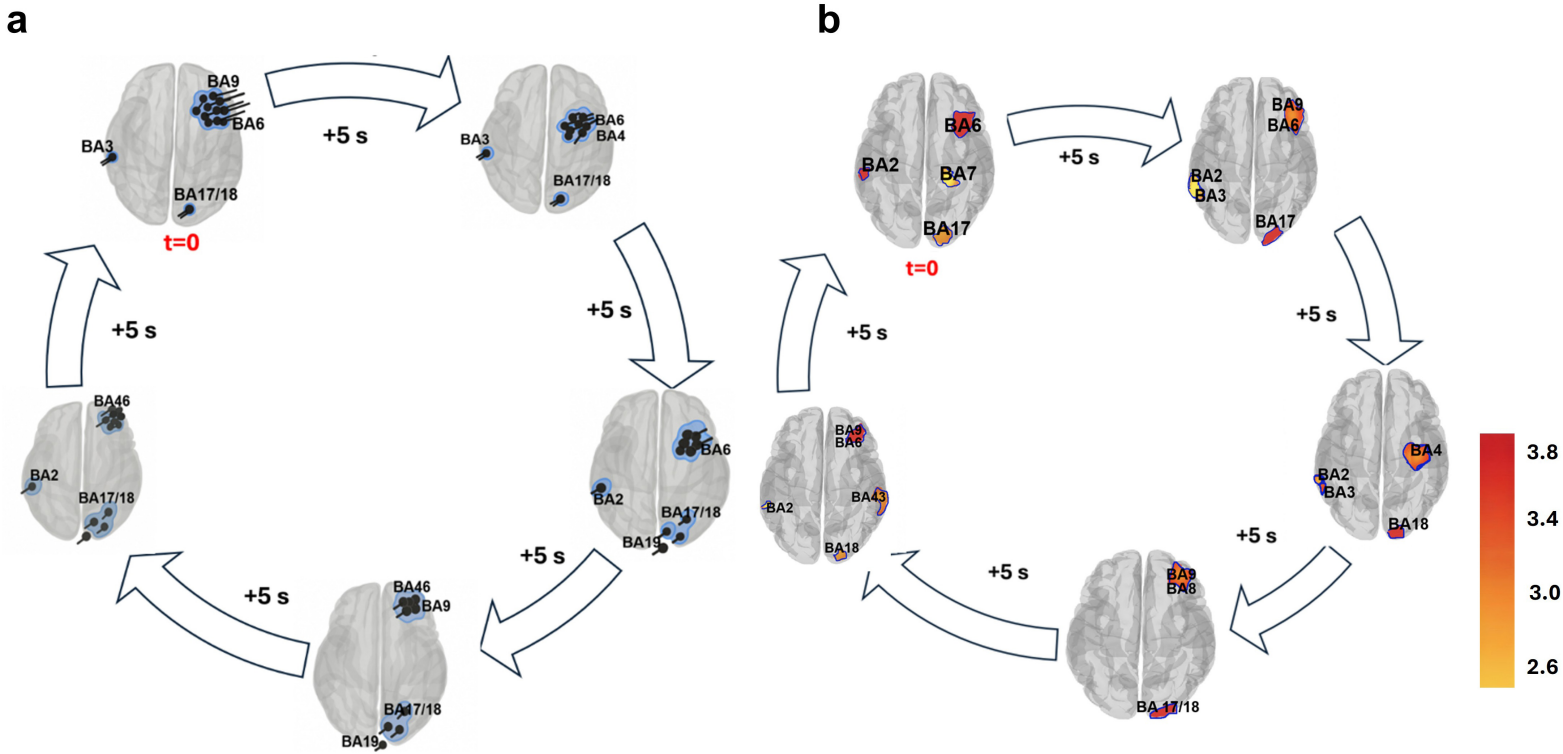
Spatiotemporal evolution of alpha-band activity over one cycle following transcranial photobiomodulation (tPBM). Time zero (0 s) marks the onset of neural recording immediately after stimulation. Sequential snapshots show the progression of cortical sources identified by dipole fitting **(a)** and standardized low-resolution electromagnetic tomography (sLORETA) **(b)**, both of which revealed structured, recurring spatial patterns that repeated with progressively lower amplitudes across five cycles. Major clusters involved prefrontal, motor, somatosensory, and visual cortices. Although specific Brodmann areas differed slightly between methods, both approaches converged on consistent network-level dynamics, indicating non-random, coordinated cortical engagement during the acute post-stimulation phase.

Figure 3 shows the evolving spatiotemporal dynamics of alpha-band activity post-tPBM, reconstructed using dipole fitting (panel a) and sLORETA (panel b). Both methods revealed a structured loop-like progression across frontal, occipital, and sensorimotor regions, repeating five times with progressively reduced amplitude until indistinguishable from background fluctuations. All sLORETA activations shown in Figure 3 represent statistically significant *t*-values obtained after 5,000-iteration permutation testing with FDR correction, and only those cortical grid points surviving this threshold are displayed.

At onset (*t* = 0 s), dipoles were localized to the dorsolateral prefrontal cortex (BA 9), premotor cortex (BA 6), early visual cortices (BA 17/18), and primary somatosensory cortex (BA 3). In parallel, sLORETA identified sources in premotor/motor regions (BA 6), prefrontal cortex (BA 9), and early visual areas (BA 17), along with contralateral somatosensory engagement (BA 2). Despite minor differences in exact Brodmann areas, both approaches converged on a fronto-occipital–sensorimotor configuration consistent with executive–visual coupling and preparatory attentional priming induced by tPBM (Wang C. et al., 2016).

Within 5 s post-tPBM, dipoles expanded posteriorly into the motor cortex (BA 4), while premotor (BA 6) and visual cortices (BA 17/18) remained stably active. Similarly, sLORETA revealed motor and premotor coactivation (BA 4, BA 6) together with visual and somatosensory regions (BA 2, BA 3, BA 17). This stage reflected early motor-executive integration layered upon sustained visual activity (Sauseng et al., 2005).

By 10 s, dipoles engaged contralateral somatosensory cortex (BA 2) and visual association cortex (BA 19), while sLORETA localized parallel activity in visual association regions (BA 18), somatosensory cortex (BA 3, BA 5), and premotor areas (BA 6). The complementary patterns indicated bilateral sensorimotor–visual coupling and deeper hierarchical processing (Babiloni et al., 2006).

At 15 s, dipoles appeared in the posterior middle frontal gyrus (BA 46) while occipital (BA 17/18, BA 19) and frontal (BA 9, BA 6) clusters persisted. Consistently, sLORETA showed activation in BA 46 and BA 9 along with visual association cortex (BA 18). Recruitment of BA 46, a central executive hub, suggested increasing engagement of attentional control and working memory (Fassbender et al., 2011; Ardila et al., 2016a).

By ∼20 s, frontal dipoles in BA 9 and BA 46 intensified alongside strong occipital activity, paralleled by sLORETA coactivation across BA 9, BA 46, BA 6, and BA 18 areas known to be part of visual and executive attention circuits (Menon and D’Esposito, 2022).

At 25 s, dipole configurations began returning toward their onset distribution and by ∼27 s, dipoles reappeared in BA 9, BA 6, and BA 17/18, closely mirrored by sLORETA sources in BA 9, BA 6, and BA 17/18. This marked the recurrence of the initial state and closure of one cycle. Across both modalities, this structured sequence recurred approximately every 27–30 s before gradually desynchronizing over the next 4 min. To quantify the spatial agreement between the two methods, a Spearman rank correlation was computed between dipole cluster occupancy and sLORETA activation magnitude across Brodmann areas. This analysis revealed a significant positive correspondence (ρ = 0.97, *p* = 0.033), shown in Supplementary Figure 2, indicating reproducible dipole trajectories across participants and strong anatomical alignment between dipole and sLORETA source estimates.

**FIGURE 4:**
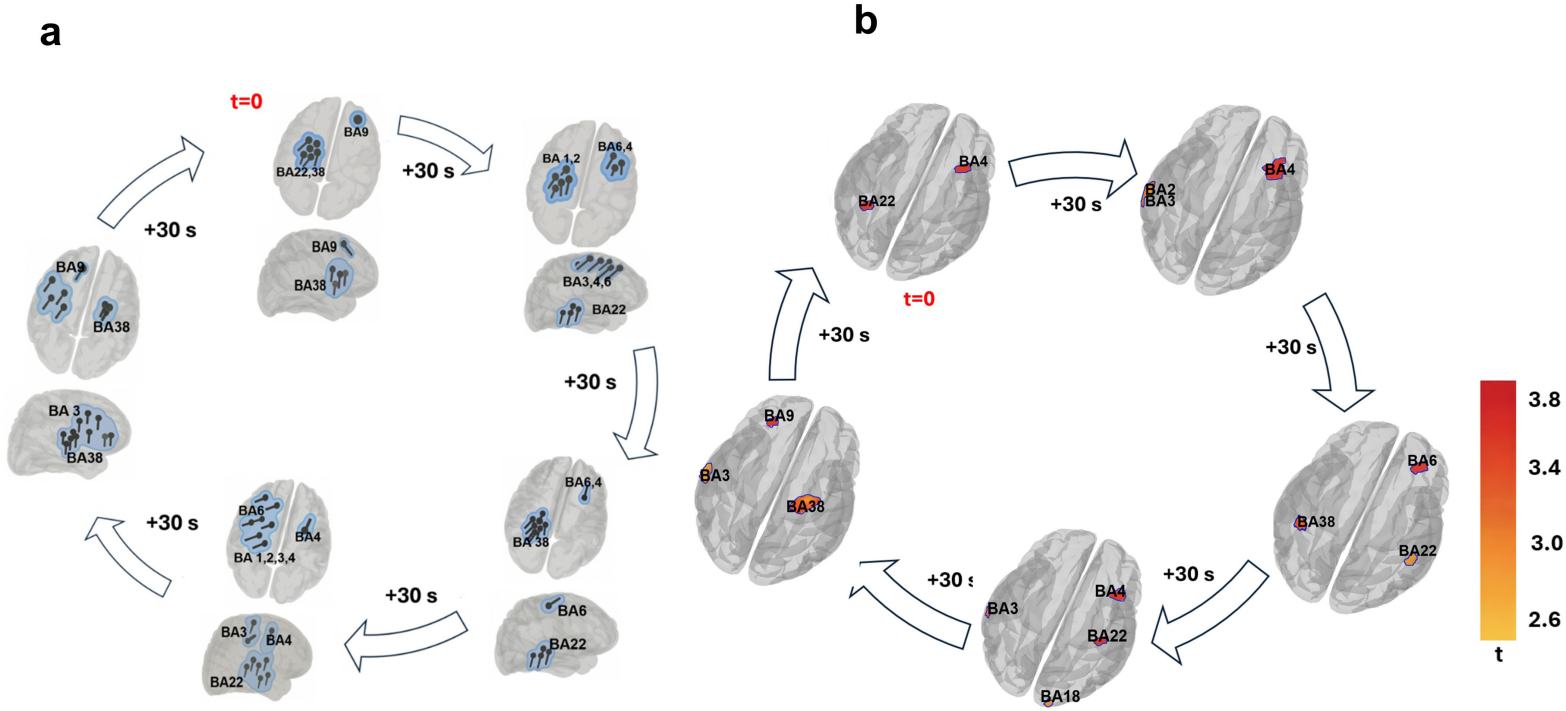
Temporally structured and spatially constrained progression of beta-band activity following transcranial photobiomodulation (tPBM). Dipole fitting **(a)** and standardized low-resolution electromagnetic tomography (sLORETA) **(b)** both revealed reproducible oscillatory trajectories across frontal, motor, somatosensory, and lateral temporal cortices, visualized at 30-s intervals over a 3-min window.

Figure 4 shows the evolving spatiotemporal dynamics of beta-band activity from (a) dipole fitting and (b) sLORETA, which occurred synchronously with the above-described alpha-band activity, immediately after the end of tPBM. All sLORETA activations shown in Figure 4 correspond to statistically significant cortical grid points that survived 5,000-iteration permutation testing with FDR correction, and only these thresholded *t*-values are displayed. To quantify spatial correspondence between modalities, we computed a Spearman rank correlation between dipole occupancy and sLORETA activation across Brodmann areas, which revealed a significant positive relationship (ρ = 0.87, *p* = 0.017),shown in Supplementary Figure 3, indicating strong anatomical agreement in beta-band source distributions.

At onset (*t* = 0 s), dipole sources localized to ipsilateral BA 9 and contralateral BA 22/38, whereas sLORETA identified activity in contralateral BA 4 and BA 22. Despite differences in exact Brodmann labeling, both approaches converged on early engagement of prefrontal–temporal and motor-associative networks, consistent with rapid post-stimulation recruitment of executive and integrative circuits (Ardila et al., 2014; Bernal et al., 2016).

By 30 s post-tPBM, dipoles advanced into ipsilateral premotor and motor cortices (BA 6, BA 4), while sLORETA showed concurrent activity in BA 6, BA 4, and BA 22/38, highlighting a broader motor–temporal integration. At 60 s, dipoles remained in ipsilateral BA 6/4 with contralateral BA 22/38, and sLORETA showed activations in the same Brodmann areas, pointing to a multimodal sensorimotor–temporal convergence (Heinrichs-Graham and Wilson, 2015; Chikermane et al., 2024).

At 90 s, dipoles shifted posteriorly into ipsilateral BA 3–4 with contralateral BA 1–3 activity; sLORETA localized parallel somatosensory and premotor regions (BA 4, BA 3, BA 43). By 120 s, both dipole fitting and sLORETA revealed anterior temporal (BA 38) and prefrontal (BA 9) re-engagement, suggesting a temporo-prefrontal coupling phase (Krishnan et al., 2018; Barone and Rossiter, 2021).

At 150 s, both methods converged on a configuration resembling the onset, with dipoles in ipsilateral BA 9 and contralateral BA 38, and sLORETA sources in overlapping frontal–temporal hubs (BA 4/9, BA 22). This recurring sequence-traversing executive, sensorimotor, and associative territories reflected a cyclic, self-organizing beta rhythm repeating with decreasing amplitude before subsiding (Krishnan et al., 2018). Supplementary material provide an animation of these dynamics (Supplementary Video 1).

**FIGURE 5:**
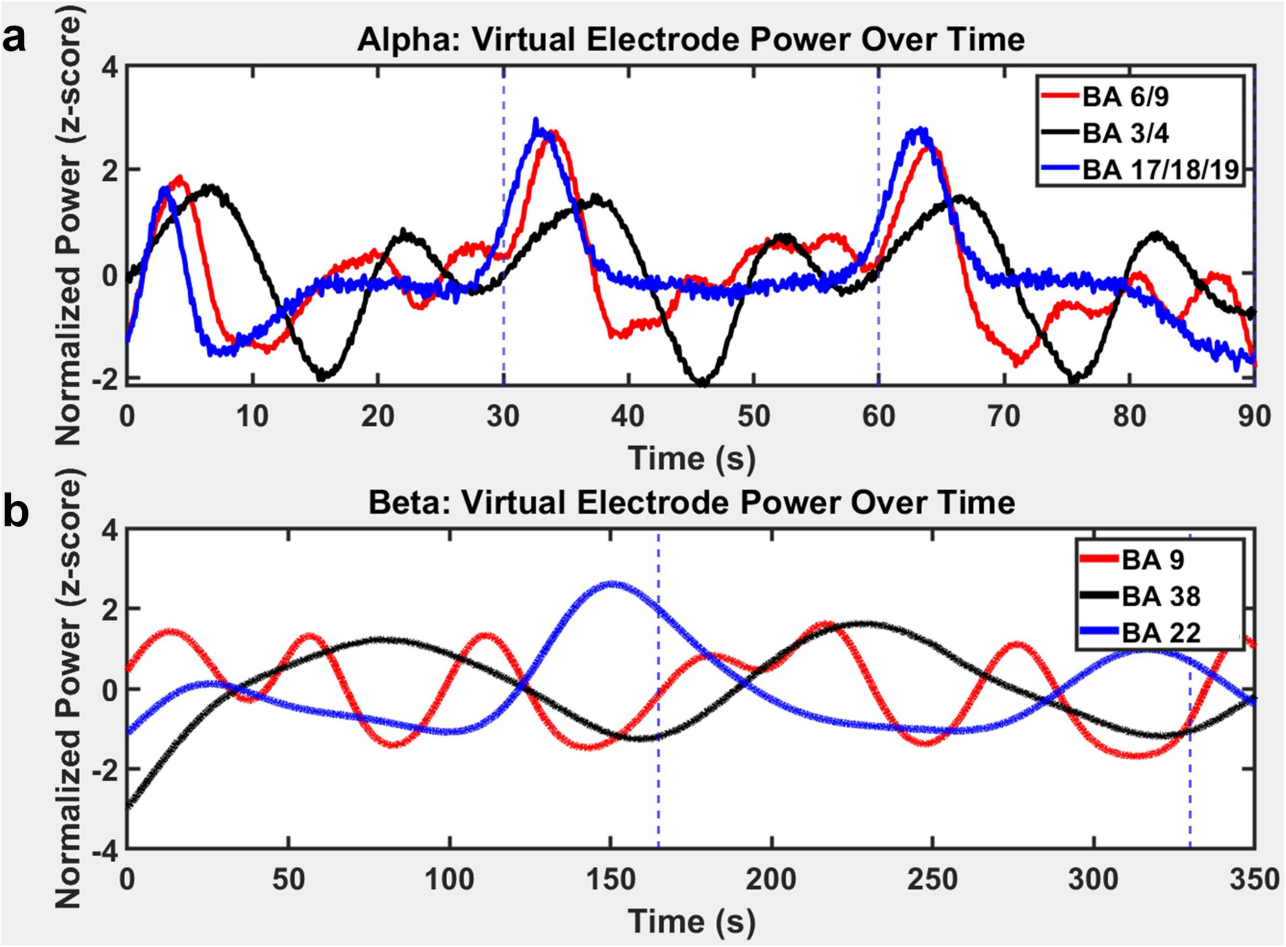
Temporally structured alpha- and beta-band power fluctuations across cortical regions following transcranial photobiomodulation (tPBM). **(a)** Normalized alpha power (z-score) power traces from virtual electrodes in prefrontal/premotor (BA 6/9), sensorimotor (BA 3/4), and visual (BA 17/18/19) cortices reveal cyclical and region-specific modulation. Power peaks appeared sequential and were often partially out-of-phase, consistent with a coordinated loop-like propagation of alpha activity across cortical networks during the early post-stimulation period. **(b)** Normalized beta power (z-score) extracted from dorsolateral prefrontal (BA 9), anterior temporal (BA 38), and superior temporal/subcentral (BA 22/43) cortices demonstrated slow, structured oscillations. A prominent anti-phasic relationship emerged between BA 22 and BA 38, where alternating peaks indicated dynamic coordination between attentional and associative processes.

To validate that the alpha-band dipoles identified were not merely static focal activations but part of a dynamic cortical process, we computed virtual electrode time-series data averaged over subjects for dipole power in individual Brodmann areas (Figure 5a). Consistent dipole power was found in the premotor and prefrontal areas (BA 6/9), sensorimotor cortex (BA 3/4), and visual cortices (BA 17/18/19). Normalized alpha power (z-scored) within each region revealed robust oscillatory fluctuations over time, with distinct but temporally structured power peaks across the cortical areas. Alpha power in visual cortex regions (BA 17/18/19) exhibited rhythmic surges approximately every 30 s, aligning with dipole loop cycles described earlier in Figure 3. In contrast, prefrontal and premotor cortical regions (BA 6/9) demonstrated alternating peaks and troughs that were often out-of-phase with sensorimotor areas (BA 3/4), suggesting coordinated but functionally distinct dynamics. This phase-shifted modulation across regions supports the view that alpha-band activity propagated as a traveling loop, engaging different nodes at different time points in a structured sequence. Likewise, to determine whether beta-band dipole activity reflected persistent activation or dynamic changes across brain regions, we extracted virtual electrode power from three sites identified in our spatiotemporal analysis: BA 9/6 (dorsolateral prefrontal and premotor cortex), BA 38 (anterior temporal cortex), and BA 22/43 (superior temporal and subcentral cortices). As shown in Figure 5b, beta power in these regions varied over time, supporting the interpretation that activity was not sustained within isolated nodes, but instead shifted rhythmically across distributed cortical areas. A key observation was the anti-phasic relationship between BA 22 and BA 38, where increases in beta power in one region were consistently accompanied by decreases in the other. This alternating pattern suggests a functional interaction between regions with distinct roles: BA 22 is associated with top-down attentional modulation (Bernal et al., 2016), while BA 38 is linked to associative and internally oriented processes (Ardila et al., 2014). Their inverse coupling does not reflect conflict, but rather a dynamic coordination between cognitive states, with one system becoming more active as the other recedes.

The observed frequencies in the relative phase of signal power between Brodmann areas, derived from source-reconstructed virtual electrodes using combined EEG and MEG data, motivated us to assess how tPBM modulates directional cortical communication. All power and connectivity estimates are reported in arbitrary units (a.u.) due to source normalization. Specifically, we computed group-level differences in directional connectivity between the tPBM and sham conditions using phase transfer entropy (PTE) for alpha and beta bands.

**FIGURE 6:**
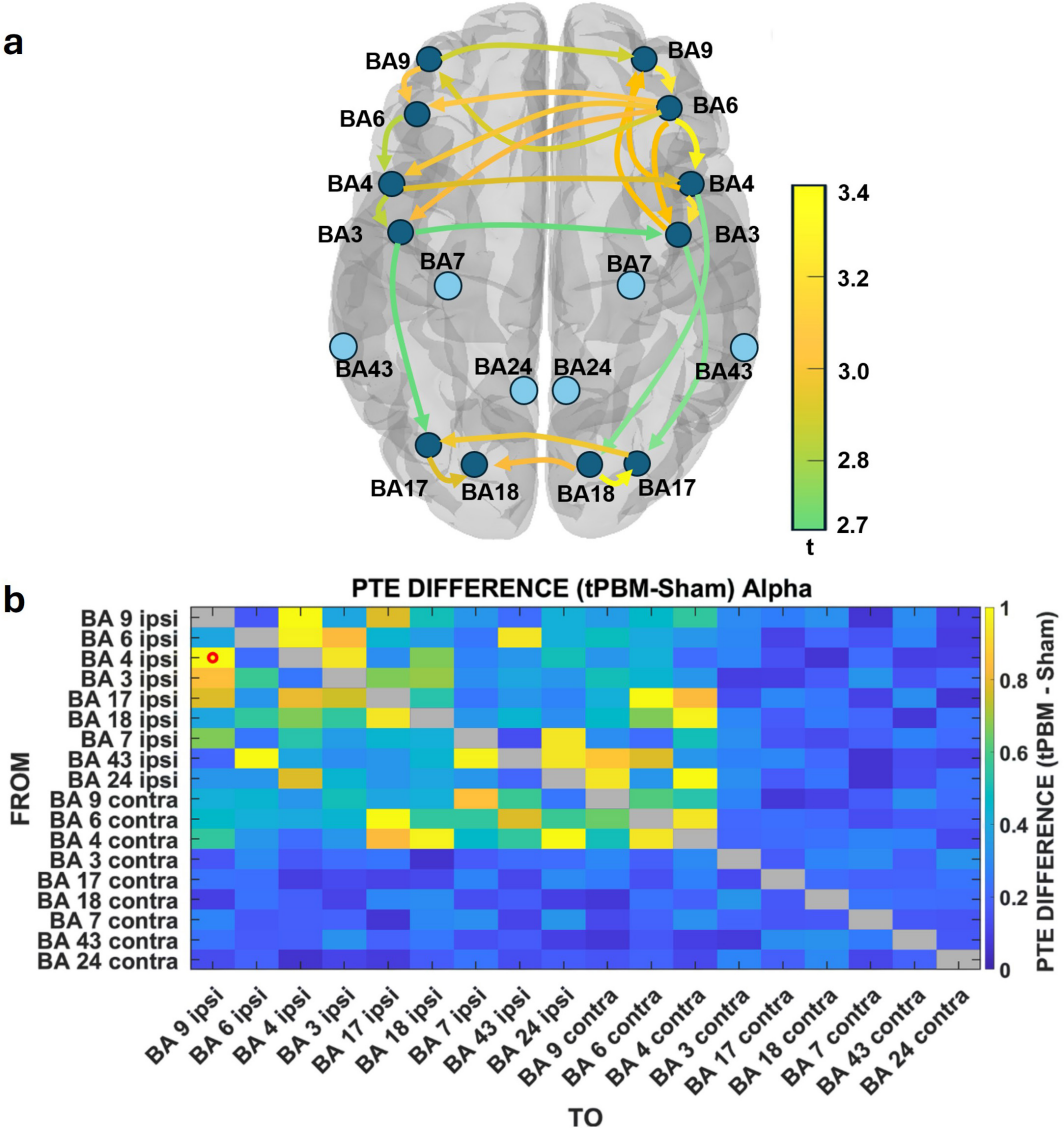
Alpha-band directional connectivity enhancements following transcranial photobiomodulation (tPBM). **(a)** Phase transfer entropy (PTE)-derived graph showing increased alpha-band information flow (tPBM – sham) from dorsolateral prefrontal cortex (BA 9) to premotor (BA 6), motor (BA 4), somatosensory (BA 3), and visual cortices (BA 17/18). **(b)** Group-averaged PTE difference matrix (z-scored) reveals strengthened intra- and interhemispheric pathways, spanning local circuits (BA 9 → 6 → 4) and long-range visual–motor connections, indicating broad alpha-band network reorganization.

Virtual electrodes were placed in cortical regions identified in the dipole analysis, including prefrontal (BA 9), premotor (BA 6), motor (BA 4), somatosensory (BA 3), and visual cortices (BA 17, 18) across both hemispheres. The resulting directed connectivity graph (Figure 6a) and PTE difference matrix (Figure 6b) reveal distinct enhancements in information flow direction in the alpha band following stimulation: We observed strong intra-hemispheric connectivity from BA 9 to BA 6, from BA 6 to BA 4, and from premotor and motor cortices to somatosensory regions (BA 3), indicating a robust frontal-to-sensorimotor propagation of alpha-band influence. Additionally, both BA 17 and BA 18 showed enhanced bidirectional connectivity, suggesting increased integration within the visual cortex. Cross-regional interactions were also evident, including premotor-to-visual, motor-to-visual, and premotor-to-somatosensory pathways, reflecting a distributed pattern of modulation consistent with global network reorganization. Notably, we also found increased ipsilateral-to-contralateral interactions, particularly in connections from left hemisphere BA 4 and BA 6 to their contralateral homologs, suggesting bilateral integration of motor and executive networks. Importantly, the connectivity edges shown in Figure 6a represent only those directional connections that survived the 5,000-iteration permutation testing with FDR correction, ensuring that only statistically significant PTE increases are visualized.

The PTE difference matrix (Figure 6b) quantifies the normalized increase in directional connectivity (tPBM – sham), with values z-scored across all connection pairs for each subject before averaging across the group. This approach highlights relative changes in network communication strength, revealing that tPBM enhanced not only local hierarchical flow (e.g., BA 9 → 6 → 4) but also long-range inter-areal coordination, particularly involving sensorimotor and visual cortex domains. To quantify the magnitude of these effects, Cohen’s *d* for the primary hierarchical pathway (BA 9 → BA 6 → BA 4) was computed, which yielded moderate-to-strong effect sizes (BA 9→6: *d* = 0.68; BA 6→4: *d* = 0.74), confirming the robustness of tPBM-induced increases in frontal-to-motor information transfer.

**FIGURE 7:**
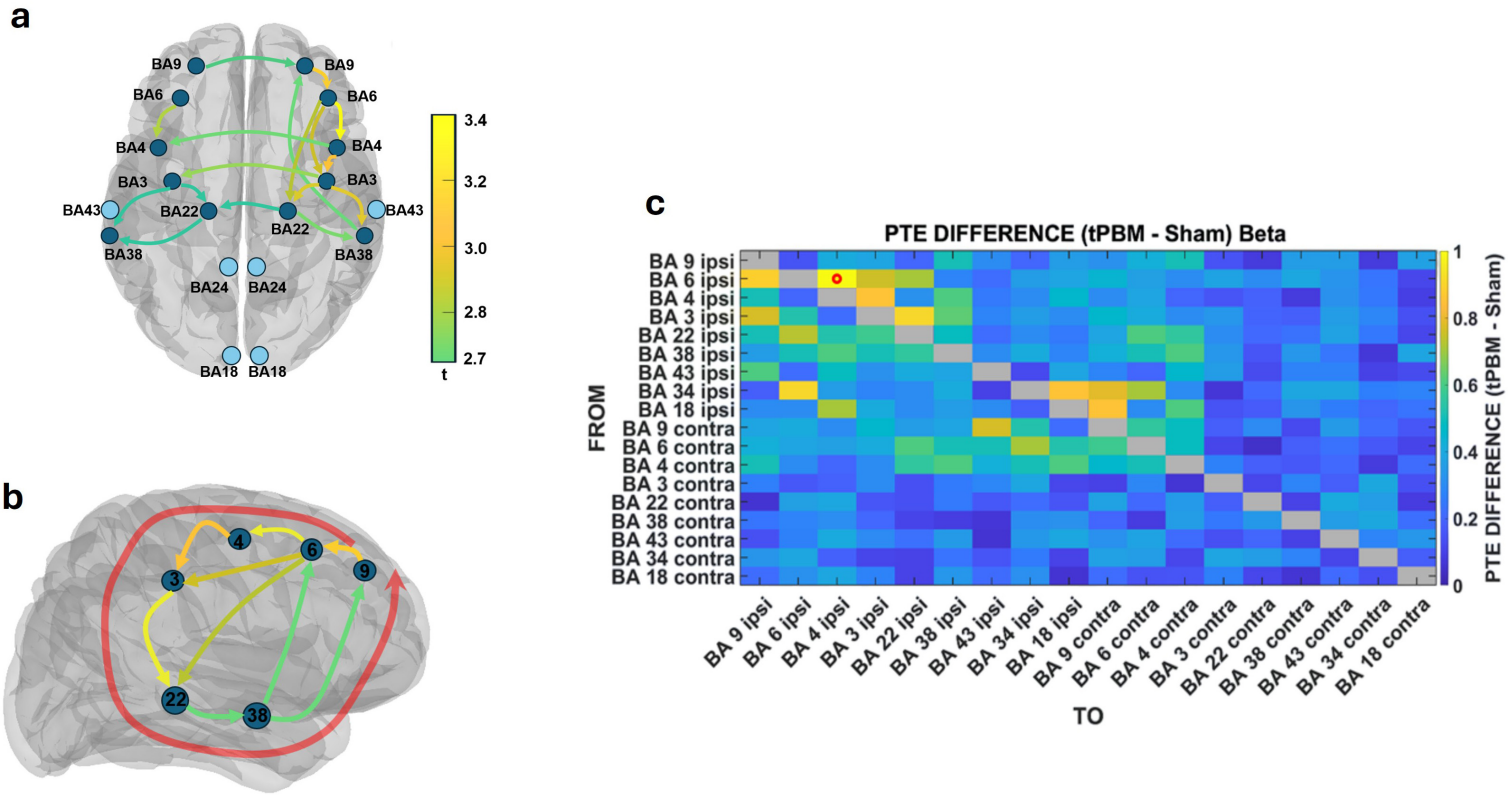
Beta-band directional connectivity increases following transcranial photobiomodulation (tPBM). **(a)** Phase transfer entropy (PTE)-derived connectivity graph showing enhanced beta-band information flow (tPBM – sham) across dorsolateral prefrontal (BA 9), premotor (BA 6), motor (BA 4), somatosensory (BA 3), and temporal cortices (BA 22, BA 38). **(b)** Sagittal schematic highlights anterior-to-posterior propagation along frontal–temporal axes. **(c)** Group-averaged PTE difference matrix (z-scored) shows strongest increases from ipsilateral BA 6 toward BA 4, BA 22, and BA 38, with bilateral projections, reflecting reorganization of executive, motor, and associative networks.

In contrast to the alpha-band’s dominant fronto-visual-sensorimotor propagation, beta-band connectivity following tPBM revealed a slower dynamic with directional flow emphasizing fronto-temporal coordination and bilateral motor network reorganization. Virtual electrodes were placed in cortical regions demonstrating strong beta-band dipole activity, including dorsolateral prefrontal (BA 9), premotor (BA 6), motor (BA 4), somatosensory (BA 3), and temporal association areas (BA 22, BA 38). The resulting directed graphs (Figures 7a,b) illustrate the organization of directional beta-band flow across these regions, while the PTE difference matrix (Figure 7c) quantifies the changes in connectivity strength between the tPBM and sham conditions.

While the canonical frontal cascade (BA 9 → BA 6 → BA 4) remained evident, the beta network uniquely engaged lateral temporal cortices, particularly BA 22 and BA 38, which were minimally involved in alpha-band interactions. These regions acted as key integrative nodes, receiving projections from premotor areas (BA 6) and contributing to extended fronto-parieto-temporal-frontal loops. Such configurations are consistent with beta’s role in internal state regulation, contextual integration, and associative processing (Chikermane et al., 2024). Additionally, interhemispheric connectivity was more prominent than in the alpha band, with ipsilateral BA 6 and BA 4 projecting to their contralateral homologs, reflecting enhanced bilateral coordination of motor-executive circuits (Heinrichs-Graham and Wilson, 2015).

The beta-band PTE difference matrix (Figure 7c) reveals a dispersed but structured increase in connectivity, with the strongest enhancements originating from ipsilateral BA 6 and targeting BA 4, BA 22, and BA 38. These patterns suggest that beta oscillations mediate slower, large-scale cortical integration, dynamically linking executive, sensorimotor, and associative hubs during resting-state periods (Barone and Rossiter, 2021; Lundqvist et al., 2024). To quantify the magnitude of these directional changes, we computed Cohen’s *d* for the primary beta-band pathways, which showed moderate-to-strong effect sizes (BA 6→4: *d* = 0.82; BA 4→3: *d* = 0.46; BA 3→22: *d* = 0.58), confirming that these beta-band interactions reflect meaningful tPBM-induced reorganization rather than noise-driven fluctuations.

To determine whether the observed spatial transitions in alpha- and beta-band dipole activity were organized by slower underlying rhythms, phase–amplitude coupling (PAC) was analyzed between infra-slow oscillations and faster EEG amplitudes. PAC significance was assessed using a surrogate-shuffle procedure, in which amplitude envelopes were circularly shifted relative to phase time series to generate a null distribution of modulation indices. The observed modulation index for each frequency pair was then z-scored, and only bins exceeding (*p* < 0.05) were considered significant. The statistically significant regions are outlined within red borders in the PAC heatmaps.

**FIGURE 8:**
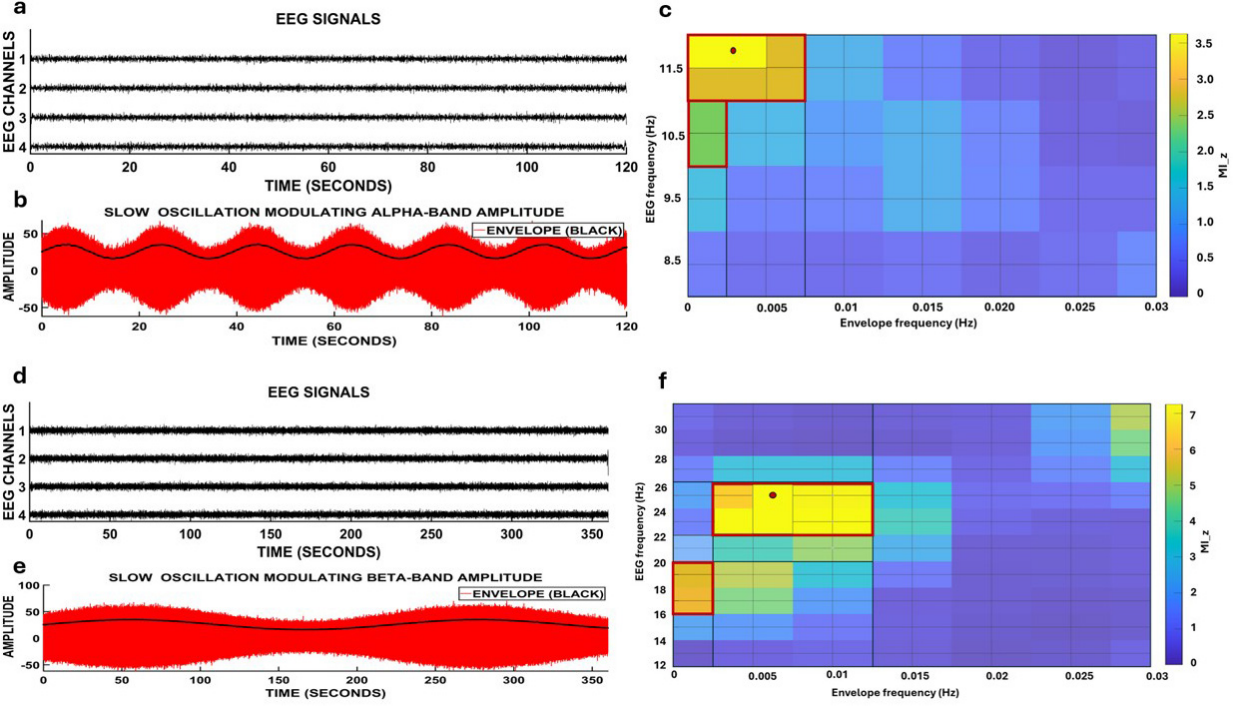
Phase–amplitude coupling (PAC) between infra-slow rhythms and faster electroencephalography (EEG) amplitudes following transcranial photobiomodulation (tPBM). **(a–c)** Show the alpha-band analysis: **(a)** raw EEG signals, **(b)** filtered alpha-band activity (8–12 Hz, red) with its slow amplitude envelope (black), and **(c)** the surrogate-shuffle–validated PAC matrix (z-scored MI), where significant coupling pairs (red borders) reveal a peak at 11.5 Hz amplitude modulated by infra-slow frequencies near 0.036 Hz. **(d–f)** Show the corresponding beta-band analysis: **(d)** raw EEG signals, **(e)** filtered beta-band activity (12–30 Hz, red) with its slow envelope, and **(f)** the statistically validated PAC matrix highlighting significant coupling near 25 Hz amplitude and 0.005–0.01 Hz envelope frequencies. Statistically significant frequency bins (*p* < 0.05) are enclosed within the red borders.

For alpha-band activity (8–12 Hz), coupling with infra-slow oscillations below 0.1 Hz was evaluated. As shown in Figure 8a, the raw EEG traces correspond to electrodes located over cortical regions that exhibited strong dipole activity in the source analysis. In Figure 8b, the bandpass-filtered alpha signal demonstrates clear amplitude modulations over time, with the overlaid black trace representing the alpha amplitude envelope. This envelope oscillates at a much slower timescale, suggesting a potential infra-slow influence. To formally assess this interaction, a surrogate-shuffle PAC analysis was performed in which amplitude envelopes were circularly shifted relative to phase signals to generate a null distribution of modulation indices. The observed values were then z-scored, and only frequency pairs exceeding (*p* < 0.05) were considered significant. The statistically significant bins are outlined with red borders in Figure 8c. The resulting PAC map revealed a clear peak centered at 11.5 Hz amplitude coupled with infra-slow frequencies near 0.036 Hz (36 mHz). Thus, the elevated PAC values in this region reflect statistically validated coupling, indicating that the slow modulation of alpha-band amplitude is structured by infra-slow rhythms rather than random amplitude fluctuations.

In parallel, we examined whether beta-band oscillations (12–30 Hz) were similarly modulated by infra-slow rhythms. PAC analysis was performed using EEG traces from electrodes positioned over regions that showed strong dipole clustering or robust source activations. As shown in Figure 8d, continuous beta activity was present throughout the recording, and the band-limited beta amplitude (Figure 8e) exhibited a slow, undulating envelope (black), suggesting the presence of ultra-slow modulatory influences. To formally test this, a surrogate-shuffle PAC analysis was applied by circularly shifting the beta amplitude envelope relative to the phase signal to create a null distribution of modulation indices. The observed modulation index values were then z-scored, and only values exceeding (*p* < 0.05) were retained. The significant frequency pairs are outlined with red borders in Figure 8f. The PAC heatmap showed a prominent and statistically significant cluster centered at ∼25 Hz amplitude coupled to infra-slow frequencies in the 0.005–0.01 Hz range, with additional structure spanning the 22–26 Hz beta window. These findings confirm that the observed beta-band amplitude fluctuations are driven by reliable cross-frequency coupling with infra-slow rhythms, rather than reflecting incidental or noisy amplitude variations (Palva and Palva, 2012; Krishnan et al., 2018; Orlowska-Feuer et al., 2021; Väyrynen et al., 2023).

Together, these results demonstrate that both alpha- and beta-band amplitude fluctuations are temporally structured by infra-slow modulatory rhythms, supporting the existence of a nested oscillatory framework that coordinates large-scale cortical dynamics after stimulation.

## 4 Discussion

Our study demonstrates that tPBM induces frequency-specific reproducible reorganization of large-scale cortical oscillatory networks, characterized by dynamic spatiotemporal structures and hierarchical modulation (Supplementary Figure 7). Unlike the sham condition, which exhibited diffuse, short-lived activation patterns (Supplementary Figure 4), suggestive of a transient placebo-driven effect (Ambrus et al., 2012; Hooyman et al., 2024), tPBM generated sustained, cyclic activation patterns reflecting coherent network engagement. tPBM engages intrinsic alpha- and beta-band systems in a temporally precise and anatomically structured manner, as evidenced by distinct patterns of dipole propagation, directional connectivity, and phase-amplitude coupling (PAC). These findings demonstrate the ability of our global optimization method for MEG/EEG dipole localization (Bastola et al., 2024) to identify tPBM-induced oscillatory patterns in the modulation of resting state cortical activity and connectivity that have not been reported previously.

### 4.1 sLORETA identifies executive network engagement

The transition from Default Mode Network (DMN) to Central Executive Network (CEN) activation suggested by the observations in our study (Figure 2) reflects a robust reorganization of cortical resource allocation with direct relevance to tPBM-induced cognitive enhancement. Before stimulation, engagement of BA 9 (dorsolateral prefrontal cortex), BA 23/31 (posterior cingulate/precuneus), and BA 32 (anterior cingulate) indicated DMN dominance, supporting self-referential thought, autobiographical memory, and internally directed attention (Raichle, 2015; Bowman et al., 2017). This configuration is typical of intrinsic resting-state activity, though persistent DMN hyperactivity has been linked to affective and anxiety disorders. After tPBM, greater recruitment of BA 10 (anterior prefrontal cortex) and BA 39 (angular gyrus) signaled a functional shift toward the CEN (Gazzaley, 2010; Daigle et al., 2022), a network central to cognitive flexibility, working memory, and attentional control. Specifically, BA 10 mediates prospective memory and high-level decision-making, while BA 39 integrates multimodal information for semantic reasoning and spatial attention. The continued activation of BA 32 across both conditions supports its role as a switching hub, consistent with its involvement in conflict monitoring and cognitive control. The anatomical distribution of these statistically thresholded regions is suggestive of a shift from areas commonly associated with DMN activity toward regions typically linked to executive-control processes. This DMN to CEN reorganization is further corroborated by the modularity-based community analyses performed (Glomb et al., 2020; Sporns et al., 2021), shown in Supplementary Figure 1. Together, this DMN-to-CEN reconfiguration suggests a neuroplastic mechanism through which tPBM shifts cortical processing from internally oriented to task-positive states, providing a plausible basis for the improvements in executive function and attentional control reported across clinical and aging populations.

### 4.2 Alpha-band activity reflects cyclic engagement of fronto-visual-somatosensory networks

The alpha-band response was characterized by a temporally recurring dipole loop originating in the dorsolateral prefrontal cortex (BA 9), proceeding through the premotor cortex (BA 6), and extending to both early (BA 17/18) and higher-order visual areas (BA 19), as well as bilateral somatosensory regions (BA 3, BA 2). The recurrence of this loop approximately every 27–30 s suggests a self-organizing spatiotemporal circuit, likely reflecting the cycling of attentional or internal monitoring states in the absence of task demands (Magosso et al., 2021). In our data, alpha-band oscillations consistently emerged in dorsolateral prefrontal cortex (BA 9) and propagated through premotor (BA 6), sensorimotor (BA 4, BA 3), and visual cortices (BA 17–19), reflecting a top-down trajectory that likely supports internal monitoring, preparatory inhibition, and sensory gating across distributed cortical networks (Fox and Raichle, 2007; Wang et al., 2019; Truong et al., 2022; Kim et al., 2023; Figure 3). Complementing these dipole-based findings, sLORETA source estimation revealed broader distributed activation spanning similar frontal, premotor, somatosensory, and visual cortices, reinforcing the observation of a cyclic fronto-occipital–sensorimotor loop. This joint prefrontal–occipital recruitment represented a peak phase of integration within a visual–executive–attention circuit, likely supporting sustained cortical reorganization (Taran et al., 2022). While sLORETA has lower spatial resolution and, given the resting-state nature of the data, may attribute activity to slightly different Brodmann areas than dipole fitting, both approaches converged on the same network-level dynamics. Together, these results underscore a robust alpha-band circuit linking executive, motor, sensory, and visual regions in a recurrent, self-organizing rhythm during the acute post-stimulation phase. This interpretation is reinforced by virtual electrode traces, which revealed out-of-phase alpha power fluctuations between visual, sensorimotor, and prefrontal regions (Figure 5), unlike activations post sham stimulation where the power traces exhibit no reproducible or cyclical temporal pattern across these regions shown in Supplementary Figure 5. The presence of alternating peaks across these regions supports a model in which alpha propagates as a slow traveling wave, engaging different cortical hubs in a cyclic sequence (Lobier et al., 2018). Such rhythmic dissociation likely reflects temporally staggered windows of excitability, with inhibitory pulsing rotating across functionally distinct modules (Ma et al., 2025).

Directional connectivity analysis confirmed that alpha-mediated information flow increased following tPBM, particularly along local hierarchical pathways– BA 9 → BA 6 → BA 4 → BA 3 and across sensory-executive systems, including premotor-to-visual and interhemispheric motor connections (Figure 6). This suggests that alpha does not merely coordinate within-domain activity but facilitates long-range integration across distributed networks involved in perception, motor readiness, and executive control (Miasnikova and Franz, 2022). Because PTE quantifies directed information flow rather than causal sequence, these findings should be interpreted as reflecting strengthened directional interactions after tPBM, while the precise temporal ordering of the loop arises from integrating PTE directionality with the sequential dipole trajectories. These directed connections, coupled with the recurrent dipole architecture, point to a rhythmic reconfiguration of fronto-occipital circuits, temporally aligned with the cyclic alpha loop observed.

Critically, this alpha-loop structure was entrained by infra-slow fluctuations in the range of 0.03–0.05 Hz (Figure 8c). Phase-amplitude coupling analysis revealed that alpha amplitude was modulated by a ∼36 mHz envelope, indicating that the emergence and recurrence of alpha peaks across the cortex were not spontaneous but governed by a slower, underlying temporal scaffold. This coupling supports the hypothesis that alpha oscillations are hierarchically embedded within a slower rhythm that paces cortical state transitions, coordinating the timing of attentional and perceptual cycles (Kropotov, 2022). The alignment of the PAC peak with the alpha-loop recurrence period (∼30 s) further suggests that the spatial propagation of alpha may be organized by these infra-slow oscillations.

Moreover, our findings indicate a dynamic interplay between the DMN and task-positive networks. Specifically, we observed alternating activation between BA 38 (temporal pole) and BA 22 (posterior superior temporal gyrus), regions associated with the DMN and language processing, respectively. BA 38 has been implicated in semantic memory, social cognition, and episodic processing, while BA 22 is involved in auditory perception and language comprehension (Langlois et al., 2010; Ardila et al., 2016b). The observed anticorrelation between these regions suggests that tPBM may facilitate the switching between internally and externally directed cognitive states, mediated by alpha oscillatory dynamics (Helfrich and Knight, 2016).

### 4.3 Beta-band dynamics reflect fronto-parietal-temporal integration and bilateral motor coordination

Whereas alpha activity exhibited cyclic fronto-visual-sensorimotor propagation, beta-band dynamics revealed a slower, spatially extended trajectory involving executive and associative networks (Figure 4). Dipole fitting (panel a) traced a fronto-parieto-temporal-frontal loop over a ∼3-min cycle, with consistent recruitment of dorsolateral prefrontal cortex (BA 9), premotor/motor regions (BA 6, BA 4), somatosensory areas (BA 3), and bilateral lateral temporal cortices (BA 22 and BA 38). sLORETA (panel b) corroborated this loop, highlighting distributed engagement of motor (BA 4, BA 6), somatosensory (BA 3, BA 43), prefrontal (BA 9, BA 8), and temporal cortices (BA 22, BA 38, BA 42). Although the exact Brodmann areas occasionally differed due to sLORETA’s lower spatial resolution, both methods converged on the same network-level dynamics. The recurrence of this trajectory approximately every ∼3 min indicates a stable, self-repeating network traversal distinct from the faster alpha recurrence. Importantly, the consistent inclusion of lateral temporal association areas-absent in the alpha trajectory-suggests that beta activity supports integration of internal cognitive processes such as semantic representation, contextual updating, and memory maintenance (Li et al., 2017; Schmidt et al., 2019). This aligns with beta’s established role in stabilizing ongoing cognitive states and supporting top-down regulation (Gazzaley and Nobre, 2012; Wang C. et al., 2016; Lundqvist et al., 2024). Furthermore, virtual electrode power traces revealed anti-phasic fluctuations between BA 22 and BA 38 (Figure 6) implying functional toggling between temporal subsystems associated with external attentional alignment (BA 22) and internally guided processing (BA 38), which was absent in post sham dipoles (Supplementary Figure 6). The structured alternation between these regions suggests that beta power may gate access to distinct processing modes on a slower-than-alpha timescale (Oostenveld et al., 2011).

Beta-band directional connectivity analysis supported this interpretation, revealing dominant projections from BA 6 toward both motor regions and lateral temporal cortices (Figure 7). The frontal beta network thus orchestrated a distributed pattern of feedforward influence linking premotor hubs to associative and sensorimotor modules. Notably, beta-mediated interhemispheric projections were more prominent than in alpha, particularly from ipsilateral to contralateral motor and premotor areas, indicating enhanced bilateral coupling in beta. This spatial configuration aligns with the proposed role of beta in maintaining network stability during internally sustained cognitive and motor states (Schmidt et al., 2019).

These slow dynamics were temporally structured by an ultra-slow modulator near 6 mHz. PAC analysis showed that beta amplitude was nested within an envelope oscillating at ∼5–6 mHz, approximately one full cycle every 2.5–3 min, which matched the recurrence interval of the beta dipole loop (Figure 8). This entrainment suggests that beta-band activity is organized by an even slower oscillatory driver that may reflect a latent cortical state cycle. Such deep infra-slow modulation likely acts as a global clock regulating the temporal deployment of fronto-parietal-temporal circuits in resting-state cognition (Popescu et al., 2024; Bastola et al., 2025).

### 4.4 Distinct temporal hierarchies structure alpha and beta network engagement after tPBM

Together, our findings reveal that tPBM induces spatially and temporally structured modulations of alpha and beta networks via distinct but complementary pathways. Alpha-band activity followed a fast, cyclic loop engaging perceptual, motor, and executive nodes, embedded within a ∼36 mHz modulating rhythm. In contrast, beta activity formed a slower, spatially expansive fronto-parietal-temporal loop entrained by ultra-slow ∼6 mHz oscillations. These nested frequency bands exhibited different propagation geometries, recurrence intervals, and functional targets-yet both were hierarchically gated by infra-slow modulators (Palva and Palva, 2012; Orlowska-Feuer et al., 2021; Väyrynen et al., 2023).

Importantly, these effects emerged in the absence of external task demands, demonstrating that tPBM can reorganize intrinsic cortical dynamics by amplifying endogenous rhythmic structures. Rather than inducing indiscriminate arousal or general activation, tPBM appears to enhance the coherence, directionality, and nested coupling of existing neural oscillations. The observed increases in alpha and beta connectivity, the emergence of stable spatial loops, and the strengthened phase-amplitude interactions all point toward a frequency-specific augmentation of hierarchical brain network function.

## 5 Conclusion

Our findings demonstrate that tPBM engages intrinsic cortical networks through structured, frequency-specific modulation of brain dynamics, revealing a novel mechanism of non-invasive neuromodulation. By integrating distributed source imaging, dipole trajectory analysis, and directed connectivity, we show that tPBM induces a reproducible shift from default mode to executive network dominance, initiates temporally recurring alpha- and beta-band oscillatory loops along anatomically defined pathways, and strengthens directional information flow across perceptual, motor, and associative regions. tPBM produces neuromodulatory effects through mitochondrial upregulation, nitric oxide–mediated vasodilation, and enhanced tissue oxygenation processes that unfold on timescales from seconds to minutes (Wang et al., 2017). The immediate post-illumination period, therefore, captures the early expression of these metabolic and neurovascular changes, making it an informative window for resolving source-level dynamics (Keszler et al., 2022). The rapid reorganization observed here likely reflects the onset of short-latency metabolic effects rather than delayed adaptations alone (Salehpour et al., 2018). These dynamics, entrained by distinct infra-slow modulators and emerging spontaneously during rest, suggest that tPBM amplifies and temporally organizes endogenous neural rhythms rather than evoking nonspecific activation. Infra-slow rhythms occur within frequency ranges associated with several well-established neurophysiological processes, including vasomotor oscillations, fluctuations in cerebral blood volume and oxygenation, and astrocytic calcium wave dynamics (Hughes et al., 2011; Watson, 2018). These slow rhythms also reflect metabolic cycling related to mitochondrial redox state and nitric-oxide–dependent vascular regulation (Kembro et al., 2014). The specific frequencies observed here ∼0.036 Hz modulating alpha and ∼0.006 Hz modulating beta, closely overlap with known vascular and neuroglial oscillatory bands and therefore plausibly represent slow neurovascular–metabolic fluctuations that are sensitive to tPBM’s bioenergetic and vasodilatory effects (He et al., 2018). This work establishes a mechanistic framework in which light-based stimulation reconfigures resting-state networks through hierarchically nested control of oscillatory activity, offering a principled foundation for precision neuromodulation. Future research should examine inter-individual variability in these spatiotemporal responses, their dependence on behavioral or cognitive state, and their translational relevance in clinical populations-ultimately enabling targeted modulation of frequency-specific network trajectories for personalized cognitive enhancement and therapy. Multimodal approaches combining MEG/EEG with concurrent near-infrared spectroscopy would further strengthen mechanistic insight by linking tPBM-induced metabolic and hemodynamic changes to the observed shifts in oscillatory activity and network connectivity. Likewise, future studies should also incorporate biologically inert control wavelengths or continuous temperature monitoring to definitively isolate photobiomodulatory effects from nonspecific thermal or placebo contributions.

## Data Availability

The raw data supporting the conclusions of this article will be made available by the authors, without undue reservation.
