# Supplementary material for: Transcranial 1064-nm laser photobiomodulation modulates frequency-specific cortical source dynamics and functional connectivity in healthy adults
Source: Front Hum Neurosci. 2026 Jan 8;19:1704482. doi: 10.3389/fnhum.2025.1704482 (PMC12823878; doi:10.3389/fnhum.2025.1704482)
Supplement: Supplementary file 1 [file Data_Sheet_1.pdf]

SUPPLEMENTARY MATERIALS

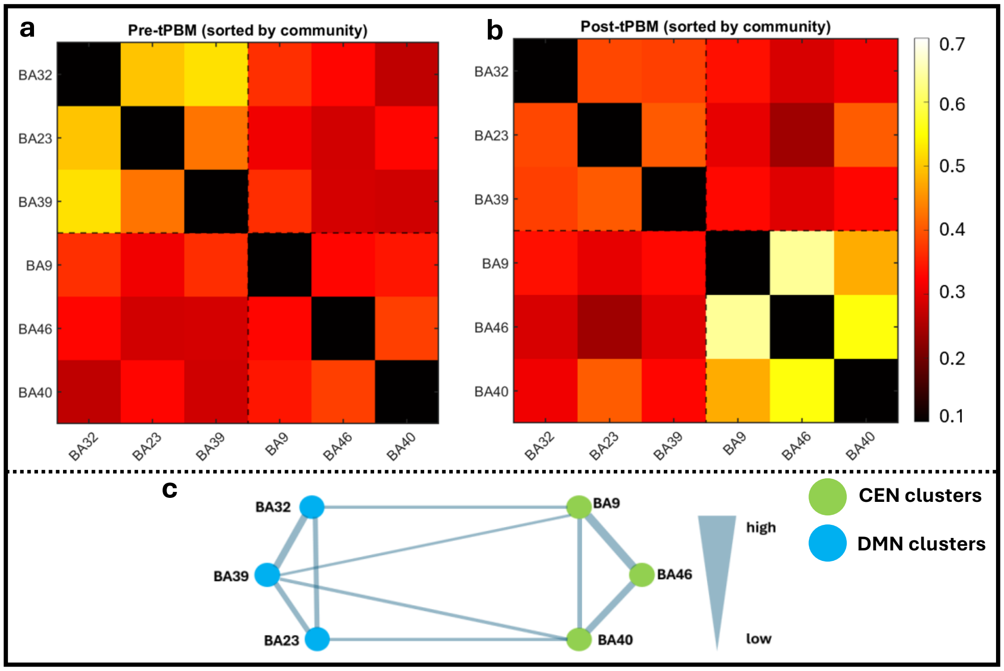

Figure S1. Graph-theoretic community analysis of virtual-electrode connectivity derived from sLORETA ROIs: (a–b) Virtual electrodes were placed at the group-level sLORETA-activated Brodmann areas and pairwise functional connectivity was computed using amplitude-envelope correlation (AEC). For each condition (pre- and post-tPBM), the resulting 6×6 connectivity matrices were arranged according to their community structure identified using Louvain modularity. Pre-tPBM, BA32–BA23–BA39 formed DMN network, whereas post-tPBM, BA9–BA46–BA40 organized into a distinct CEN-like module, demonstrating clear separation between the two canonical networks. (c) To assess the stability of the detected community assignments, Louvain modularity was repeated 1,000 times with randomized initializations. For each pair of ROIs, we computed a co-assignment index indicating the proportion of runs in which the nodes were placed in the same community. Edge thickness reflects this co-assignment tendency: thicker edges denote pairs of regions that consistently clustered together across Louvain runs. The post-tPBM configuration shows robust within-module associations among CEN-related regions (BA9–BA46–BA40) and similarly strong cohesion among DMN-related regions (BA32–BA23–BA39), further supporting the emergence of distinct DMN and CEN network structure following tPBM.

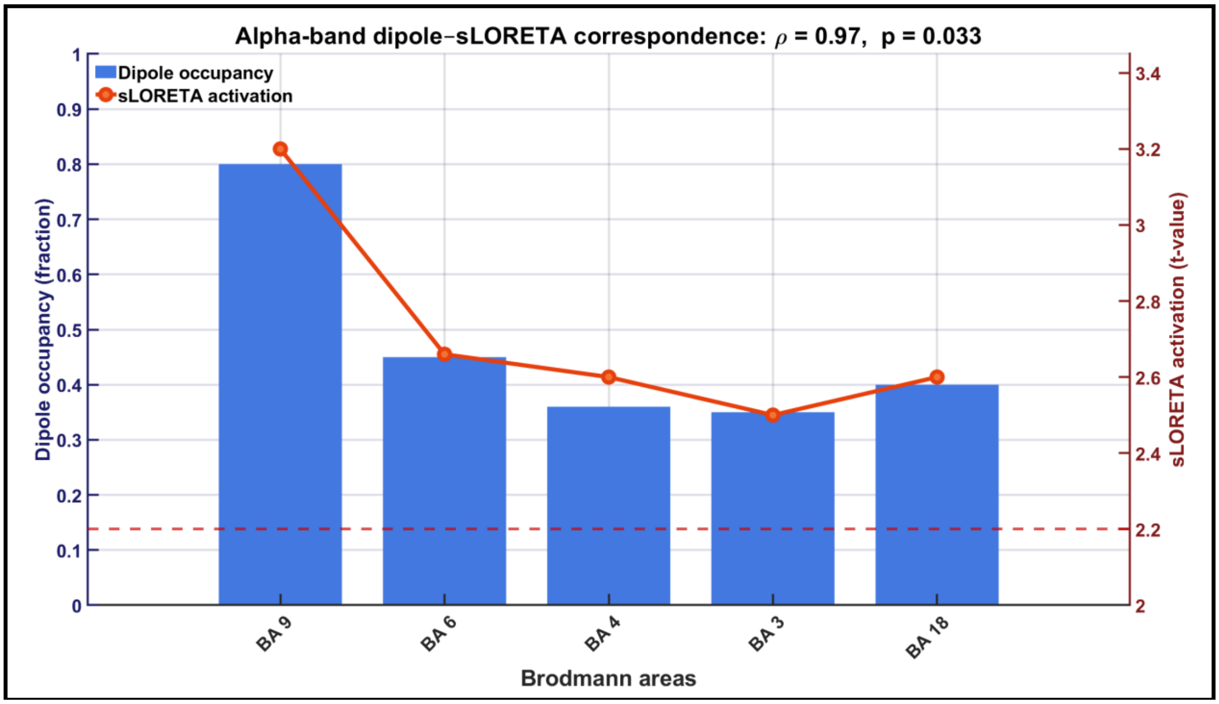

Figure S2. Dipole–sLORETA spatial correspondence for the alpha band.

Spearman rank correlation was computed between (1) the dipole occupancy in each Brodmann area (i.e., the fraction of all localized dipoles falling within a given ROI at the analyzed time point) and (2) the corresponding sLORETA activation magnitude in the same ROIs. The analysis revealed a strong positive correlation (ρ = 0.97) that reached statistical significance (p = 0.033), indicating that regions containing a higher proportion of localized dipoles tended to exhibit stronger sLORETA activation. Statistical significance for Spearman ρ was computed using the null hypothesis that no monotonic association exists between the two ranked variables. Red dashed line denotes the threshold above which sLORETA activations survived FDR correction.

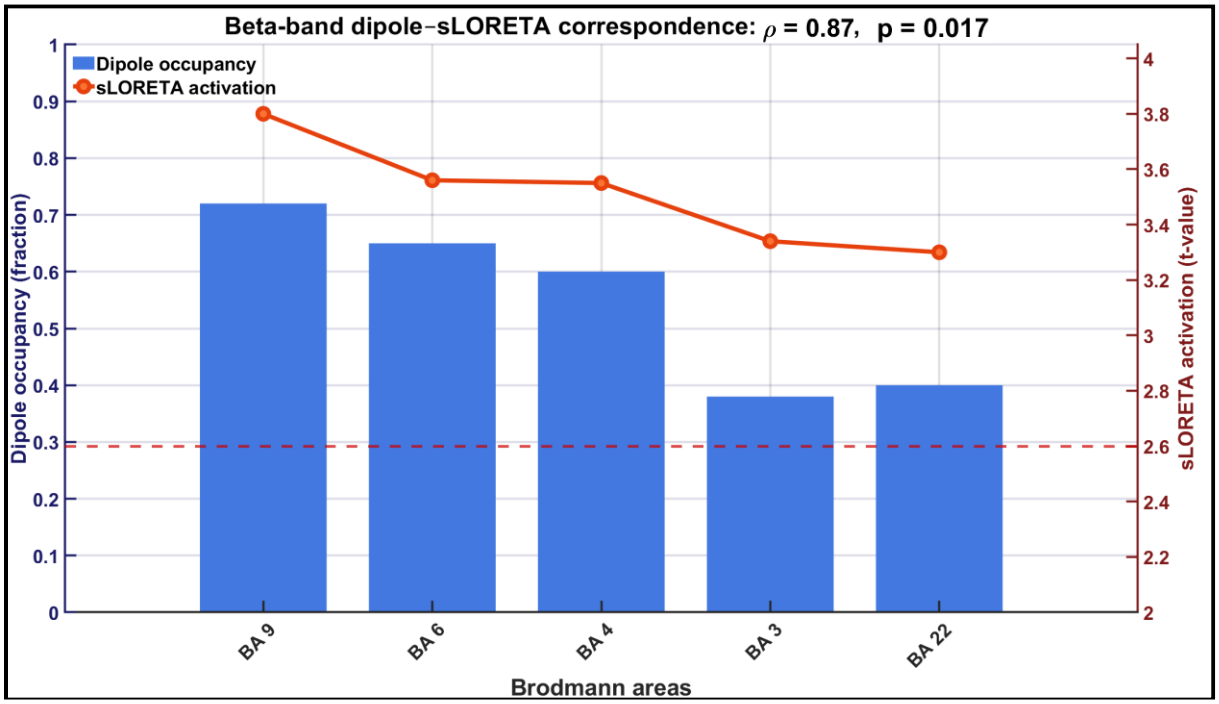

Figure S3. Dipole–sLORETA spatial correspondence for the beta band.

Spearman rank correlation was computed between (1) the dipole occupancy in each Brodmann area (i.e., the fraction of all localized dipoles falling within a given ROI at the analyzed time point) and (2) the corresponding sLORETA activation magnitude in the same ROIs. The analysis revealed a positive correlation ($\rho = 0.87$) that reached statistical significance ($p = 0.017$), indicating that regions containing a higher proportion of localized dipoles tended to exhibit stronger sLORETA activation. Statistical significance for Spearman ρ was computed using the null hypothesis that no monotonic association exists between the two ranked variables. Red dashed line denotes the threshold above which sLORETA activations survived FDR correction.

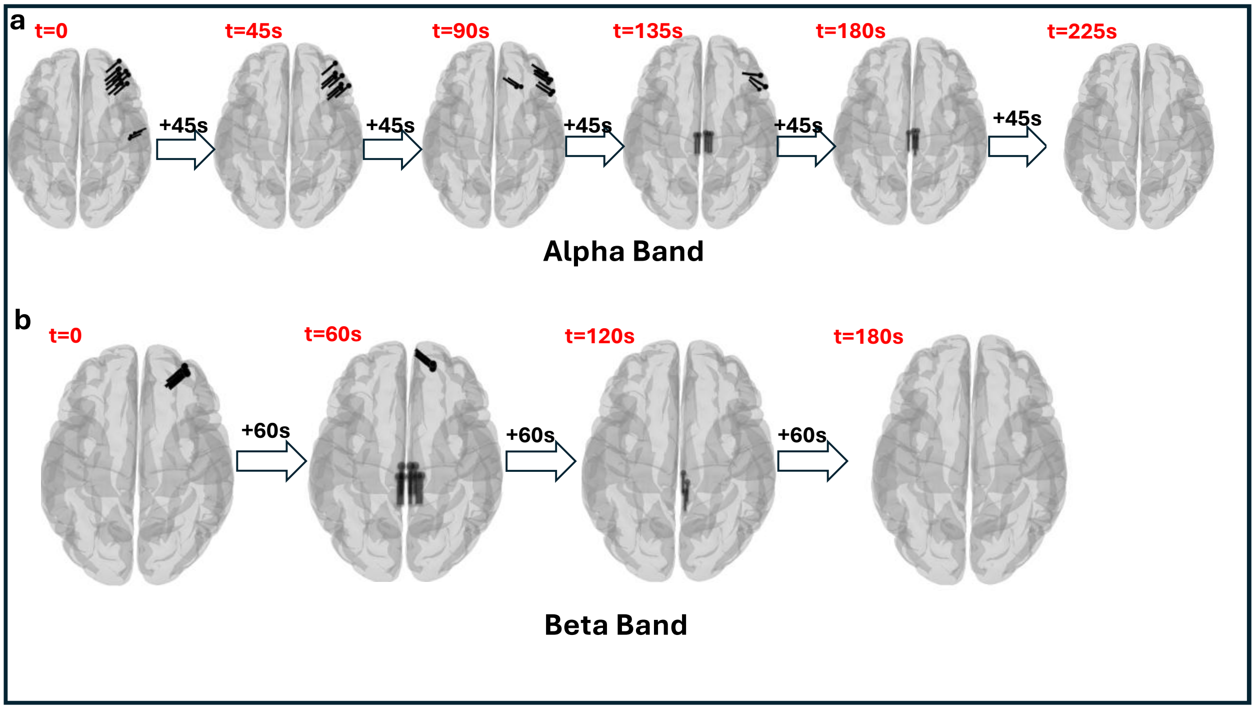

Figure S4. Post-sham stimulation dipole propagation dynamics in the alpha and beta bands. (a) Alpha band: Dipole activations from post-sham MEG/EEG data show an initial transient activation but fail to exhibit the organized, repeating propagation loops observed after tPBM. Instead, dipole trajectories disperse irregularly across time, with no evidence of cyclical re-engagement or stable directionality. Activity gradually diminishes, indicating a non-sustained and non-cyclical pattern of cortical propagation.(b) Beta band: Similarly, beta-band dipole activity following sham stimulation shows brief, spatially inconsistent discharges that fade rapidly over time. No coherent propagation sequence or recurrent pathway emerges, contrasting sharply with the robust, loop-like propagation patterns identified in the post-tPBM condition. Overall, sham stimulation produces weak, non-periodic dipole dynamics that dissipate rather than forming structured cortical loops.

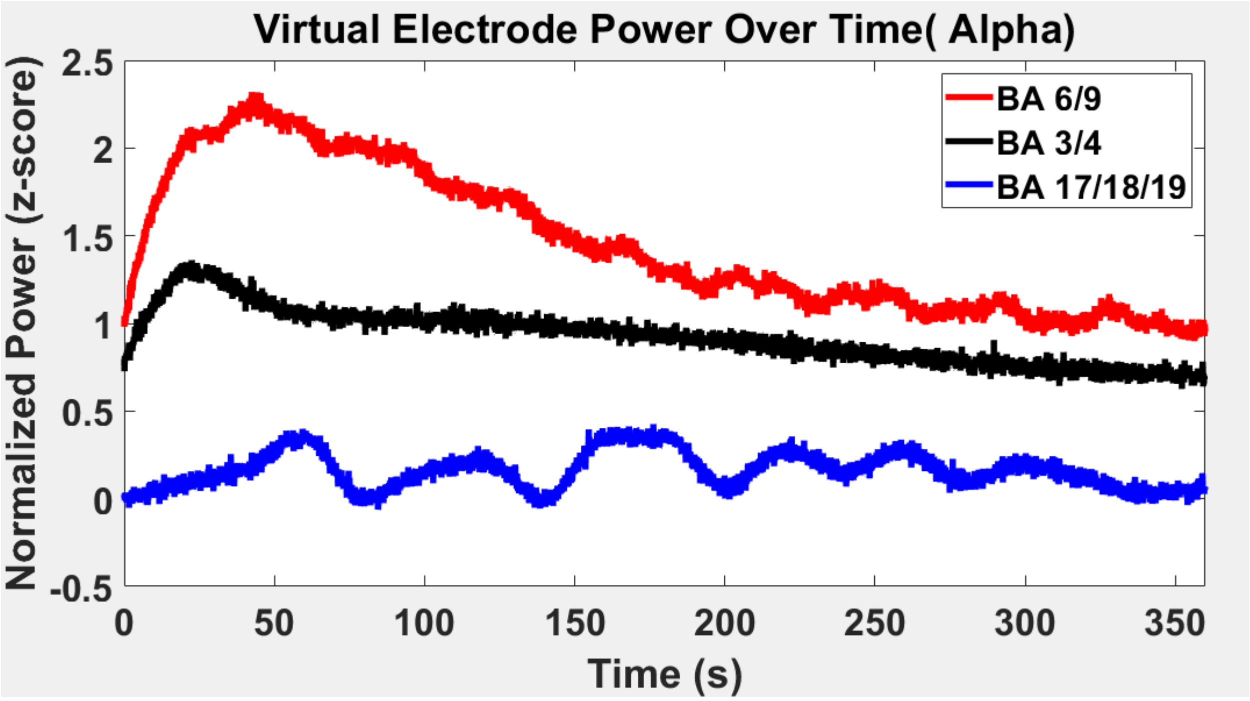

Figure S5. Virtual electrode power dynamics following sham stimulation (alpha band). Virtual electrodes were placed in the same regions of interest– the prefrontal/premotor (BA 6/9), sensorimotor (BA 3/4), and visual cortices (BA 17/18/19)– corresponding to the hubs that showed significant activation patterns in the post-tPBM data. Unlike the temporally structured and cyclical alpha-band modulations observed after tPBM, the post-sham traces show no consistent rhythmic or repeating patterns in any of the examined regions. The gradual rise and decay of power likely reflects nonspecific or placebo-related fluctuations, which diminish over time and do not exhibit the organized propagation dynamics characteristic of the true tPBM response.

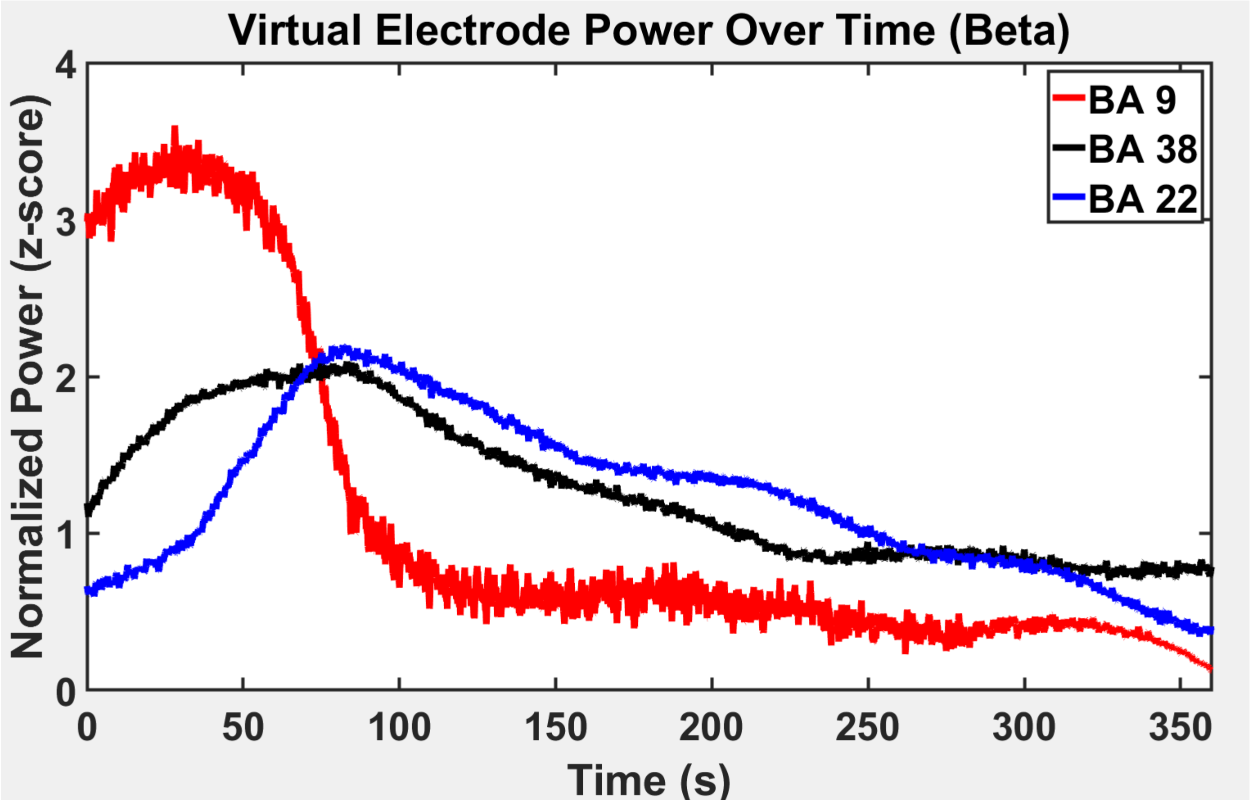

Figure S6. Virtual electrode power dynamics following sham stimulation (beta band). Virtual electrodes were placed in the same beta-band hubs identified in the post-tPBM analyses: dorsolateral prefrontal cortex (BA 9), superior temporal cortex (BA 22), and anterior temporal cortex (BA 38). In contrast to the structured, sequential beta-band propagation observed after tPBM, the post-sham power traces exhibit no reproducible or cyclical temporal pattern across these regions. The observed fluctuations likely reflect nonspecific drifts or placebo-related transients that gradually diminish over time and do not display coordinated inter-regional dynamics. This absence of organized temporal structure underscores the specificity of the propagation patterns detected following true tPBM exposure.

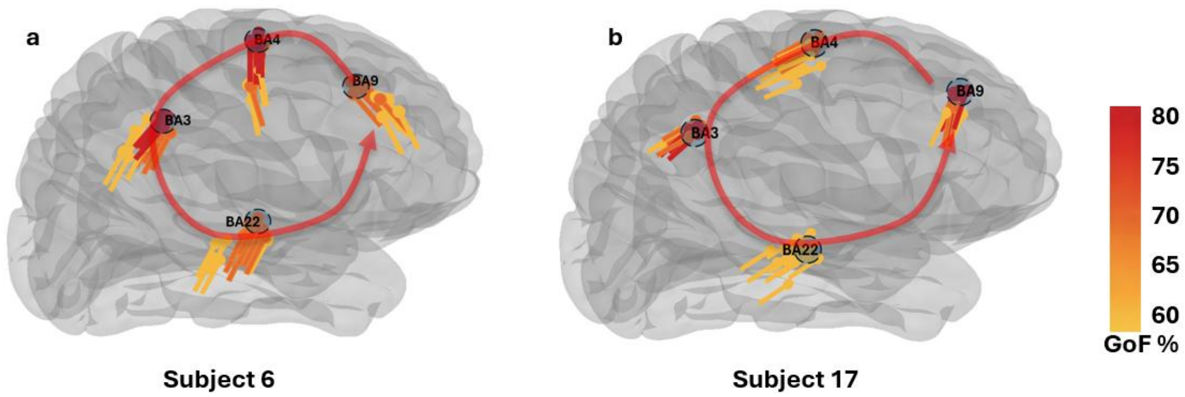

Figure S7. Sagittal view of beta-band dipole clusters for two representative subjects. Sagittal cortical renderings illustrate the beta-band dipole localizations for (a) Subject 6 and (b) Subject 17. Dipoles with goodness-of-fit (GoF = 1 – residual variance) greater than 60% were retained and overlaid on the cortical surface to highlight spatially consistent hubs of activation that emerge across the propagation sequence. The schematic shows a clear anterior-to-posterior propagation trajectory along frontal–temporal pathways along consistent Brodmann areas, and dipoles with greater goodness-of-fit exhibit tighter clustering across these regions.
